# Impact of Preinjury Antithrombotic Therapy on 30–Day Mortality in Older Patients Hospitalized With Traumatic Brain Injury (TBI)

**DOI:** 10.3389/fneur.2021.650695

**Published:** 2021-05-13

**Authors:** Pål Rønning, Eirik Helseth, Ola Skaansar, Cathrine Tverdal, Nada Andelic, Rahul Bhatnagar, Mathias Melberg, Nils Oddvar Skaga, Mads Aarhus, Sigrun Halvorsen, Ragnhild Helseth

**Affiliations:** ^1^Department of Neurosurgery, Oslo University Hospital Ullevål, Oslo, Norway; ^2^Faculty of Medicine, University of Oslo, Oslo, Norway; ^3^Department of Physical Medicine and Rehabilitation, Oslo University Hospital, Oslo, Norway; ^4^Department of Cardiology, Oslo University Hospital Ullevål, Oslo, Norway; ^5^Department of Pulmonology, Oslo University Hospital Ullevål, Oslo, Norway; ^6^Department of Anesthesiology, Oslo University Hospital, Oslo, Norway

**Keywords:** traumatic brain injury, antithrombotic therapy, antiplatelet therapy, anticoagulant therapy, elderly, mortality

## Abstract

**Objective:** Elderly patients are frequently in need of antithrombotic therapy for reducing thrombotic events. The association between antithrombotic drugs and survival after traumatic brain injury (TBI) is, nevertheless, unclear.

**Methods:** This retrospective study included patients ≥65 years admitted to a Norwegian Level 1 trauma center with TBI identified on cerebral computed tomography (cerebral-CT) during 2014–2019. Preinjury use of antiplatelets and anticoagulants was compared to the prescription rate in the general Norwegian population. The primary outcome was 30-day mortality. Uni- and multivariate logistic regression analyses estimated the association between the use of antithrombotic drugs and mortality.

**Results:** The study includes 832 consecutive TBI patients ≥65 years. The median age was 76 years, 58% were males, 51% had moderate or severe TBI, and 39% had multiple traumas. Preinjury use of antithrombotics was registered in 471/832 (55.6%) patients; antiplatelet therapy alone in 268, anticoagulant therapy alone in 172, and combined antiplatelet and anticoagulant therapy in 31. Antiplatelet use did not differ between the study cohort and the general Norwegian population ≥65 years (31 vs. 31%, *p* = 0.87). Anticoagulant therapy was used more commonly in the study cohort than in the general Norwegian population (24 vs. 19%, *p* = 0.04). Combined use of antiplatelet and anticoagulant therapy was significantly associated with 30-day mortality, while preinjury antiplatelet or anticoagulation treatment alone was not. No difference in 30-day mortality between patients using VKA, DOACs, or LMWH was encountered.

**Conclusions:** In this cohort, neither antiplatelet nor anticoagulant therapy alone was associated with increased 30-day mortality. Anticoagulant use was more prevalent among TBI patients than the general population, suggesting that anticoagulation might contribute to the initiation of intracranial bleeding after blunt head trauma. Combined antiplatelet and anticoagulant therapy posed increased risk of 30-day mortality.

## Introduction

Antithrombotic therapy, comprising antiplatelet and anticoagulant drugs, is frequently used in the elderly population. Antiplatelet drugs impair the migration and aggregation of platelets, while anticoagulants inhibit the blood's ability to clot by inhibiting distinct proteins in the coagulation cascade. According to The Norwegian National Prescription Registry in 2019 (1), about one-third and one-fifth of people in Norway ≥65 years old were prescribed antiplatelet drugs or anticoagulants, respectively. Established cardiovascular disease (CVD) or primary prevention against CVD are the most common antiplatelet therapy indications. Anticoagulants are most often prescribed for stroke prevention in atrial fibrillation (AF), treatment of venous thromboembolism (VTE), or prosthetic valve thrombosis prevention in patients with mechanical heart valve prostheses. The benefit of antiplatelet therapy as secondary prevention of new vascular events in patients with established CVD is thoroughly documented, as is the use of anticoagulation for the indications mentioned above (2–7). The most commonly prescribed antiplatelets in Norway are acetylsalicylic acid and the P2Y12 inhibitors. Vitamin K antagonists (VKAs), direct oral anticoagulants (DOACs), and low molecular weight heparins (LMWH) are the most commonly prescribed anticoagulants (1).

Although antithrombotic therapy reduces the risk of ischemic events in patients with CVD, AF, and VTE, they are associated with an increased risk of spontaneous and traumatic intracranial bleeding (8–13). Nevertheless, the effect of these drugs on survival and functional outcome after traumatic brain injury (TBI) is unclear. In some studies, preinjury single antiplatelet use was associated with a slightly increased risk of death after a blunt head injury, while others report no increased risk of death (12, 14–16). Anticoagulation in patients suffering a blunt head trauma appears to be associated with an increased risk of death (12, 15–18). However, even this alleged fact has recently been questioned (19, 20). Most reports date back when VKA was the dominating anticoagulant drug. Contemporary TBI series after the introduction of DOACs and P2Y12 inhibitors are needed to evaluate the current risk of preinjury antithrombotic use in TBI patients.

The aims of this study were two-fold. First, to describe the frequency of antithrombotic drug use in elderly, hospitalized patients with TBI compared to the general elderly Norwegian population. Second, to assess the association between preinjury antithrombotic therapy and 30-day mortality. Data are from Oslo University Hospital Ullevål (OUH-U).

## Methods

This study uses data from the “Oslo TBI Registry—Neurosurgery,” a prospective quality control database run by the neurosurgical department at OUH (21). The Registry uses the Medinsight database platform under the approval of the OUH Data Protection Officer (DPO approval number 2016/17569).

### Study Population

Patients ≥65 years with TBI identified on cerebral computed tomography (cerebral-CT) admitted to OUH-U between 01.07.2014 and 31.12.2019 were included. OUH-U is the only Level I trauma center with neurosurgical service for the South-Eastern Norway Regional Health Authority, serving 3.0 million people within a geographic area covering 110,000 km^2^. Patients exhibiting a potentially severe injury with transport time <45 min, or in need of neurosurgical care regardless of transport time, are transported directly to OUH-U. OUH-U also serves as the local acute care hospital for most of the population in Oslo. Patients not qualifying for direct transport to OUH-U are initially treated at other acute care hospitals in the region and transferred to OUH-U when needed. TBI was categorized according to Head Injury Severity Score (HISS) into minimal [Glasgow Coma Scale Score (GCS) 15 and no loss of consciousness or amnesia], mild [GCS 14 or 15 plus amnesia, or brief (<5 min) loss of consciousness, or impaired alertness or memory], moderate [GCS 9–13 or loss of consciousness ≥5 min or focal neurological deficit] or severe (GCS ≤ 8) (22). In the analysis, minimal TBI with traumatic findings on CT was grouped with mild TBI. Minimal/ mild TBI with traumatic findings on CT is by some authors referred to as complicated mild TBI (23, 24). The Rotterdam CT score was used to classify the severity of intracranial injury by CT findings and was assessed on the initial cerebral-CT with a range from 1 to 6 (worst score = 6). The score is based on (i) status of basal cisterns (normal, compressed, or absent); (ii) midline shift (0–5 or >5 mm); (iii) epidural hematoma (present or absent); (iv) traumatic subarachnoid hemorrhage/intraventricular hemorrhage (present or absent). Increased Rotterdam CT score correlates with increased mortality in patients with severe and moderate TBI (25).

Prescription data on antithrombotic therapy in the general, elderly Norwegian population were retrieved from the National Prescription Database, run by the Norwegian Institute of Public Health (www.norpd.no) (1).

### Oslo TBI Registry—Neurosurgery

The “Oslo TBI Registry—Neurosurgery” includes TBI patients fulfilling the following criteria: Traumatic brain injury with cerebral-CT showing signs of acute trauma (hemorrhage, fracture, traumatic axonal injury, vascular injury), admitted to OUH-U as an in-patient within seven days after injury, and having a Norwegian social security number. For this study, the data extracted from the registry were age, sex, pre-injury ASA score (American Society of Anesthesiologists Physical Status Classification system), living status at the time of injury (home—care for self, home—with assistance or institutionalized), preinjury antiplatelet medication (acetylsalicylic acid, clopidogrel, dipyridamole, ticagrelor, prasugrel), preinjury anticoagulation [VKA (warfarin—the only VKA used in Norway), LMWH (dalteparin, enoxaparin) and DOACs—dabigatran, apixaban, rivaroxaban, edoxaban)], indications for preinjury anticoagulation/antiplatelet therapy, injury energy (high vs. low), HISS (22), Rotterdam CT-score (25), multiple trauma (no/yes), and 30-day mortality.

### General TBI Management at OUH-U

OUH-U has 24-h neurosurgical and neuro-intensive care service. Included patients were managed according to Brain Trauma Foundation guidelines, with minor local adjustments including a protocol for complete or partial reversal of antithrombotic medication in the emergency room (Table 1) (26)^1^. Briefly, the goals of TBI treatment have been to maintain intracranial pressure (ICP) <22 mmHg and the cerebral perfusion pressure (CPP) >60 mmHg for adults. A staircase protocolled approach is employed to fulfill the ICP and CPP goals; immediate surgical evacuation of traumatic intracranial mass lesions, adequate patient positioning, suitable sedation, proper ventilator adjustments, hyperosmolar therapy, controlled normothermia, cerebrospinal fluid (CSF) drainage, and decompressive craniectomy.

**Table 1:** Protocol for complete or partial reversal of antithrombotic drugs at OUH-U following trauma or TBI with severe bleeding.

**Drug to be reversed**	**Prerequisites**	**Agents**	**Doses**
Vitamin K antagonist (VKA)	1. Confirm drug indication 2. Take adequate coagulation tests^*^	Prothrombin complex concentrate (PCC)^**^	According to matrix for body weight vs. INR, or 30 IU/kg iv.
		Vitamin K1^**^	5 mg iv.
Antiplatelet drugs	1. Confirm drug indication 2. Define when last dose was administered 3. Take adequate coagulation tests^*^	Desmopressin^**^ Tranexamic acid^**^ Add appropriate platelet transfusion^**^	0.3 mcg/kg iv. 10 mg/kg iv. 1–2 units of 350 ml (360–720 × 10^9^ platelets)
Direct oral anticoagulants (DOACs)	1. Confirm drug indication 2. Define when last dose was administered 3. Take adequate coagulation tests^*^	Reversal of dabigatran: - Idarucizumab^***^	5.0 g iv.
		Reversal of apixaban, rivaroxaban, edoxaban: - Prothrombin complex concentrate (PCC)^**^	If <15 h since last dose: 30 IU/kg iv. If 15–24 h since last dose: 20 IU/kg iv.
		- Tranexamic acid^**^	10–20 mg/kg iv.
		- Andexanet alfa	Not available for use in Norway
Low molecular weight heparin (LMWH)	1. Confirm drug indication 2. Take adequate blood samples	Protamine sulfate^**^	If <12 h since last dose: 1 mg iv. per 100 IU dalteparin or per mg of enoxaparin. If 12–24 h since last dose: 0.5 mg iv. per 100 IU dalteparin or per mg of enoxaparin.

*Vitamin K antagonists (VKA) can be completely reversed by prothrombin complex concentrate (PCC) within minutes. Antiplatelet drugs can be reversed partially. Dabigatran (NOAC) can be reversed completely by Idarucizumab within minutes, while the other NOACs can be partly reversed by PCC and Tranexamic acid. Low molecular weight heparin (LMWH) can be partially reversed by Protamine sulfat. The potential benefit of reversal is weighed against any potential negative effect on an individual basis. The presence of mechanical heart valves (particularly in the mitral position) and recent stent implantation in major coronary vessels (left main stem, left anterior descending artery (LAD), bifurcation stenting) in particular merit careful consideration as acute heart valve thrombosis and thrombotic occlusion of proximal coronary vessels have high mortality rates*.

* *INR, partial thromboplastin time (APTT), platelets, fibrinogen*.

** *Available during the whole study period*.

*** *Available in Norway from January 1, 2016*.

### Ethics

The Data Protection Officer (DPO) at OUH approved the study as a quality control study (approval number 2017/3904).

### Statistics

Data were summarized with medians and interquartile range for continuous variables, whereas we report frequencies and percentages for categorical variables. Comparisons between groups and their associated *p*-values were calculated using the non-parametric Wilcoxon rank-sum test for continuous variables. For categorical variables, the Chi-square test for independence or the exact Fisher test in small expected cell counts. Uni- and multivariate logistic regression models were fitted in order to estimate the different variables' effect on 30-day survival. A separate logistic analysis investigating the impact of the other anticoagulants on 30-day survival was also calculated. R v4.0.1 was used for all statistical analyses^2^. *P*-values <0.05 were considered significant.

## Results

This study included 832 consecutive TBI patients ≥65 years admitted to OUH-U, 830 with blunt trauma and 2 with penetrating trauma. The median age was 76 years (IQR 70–83), 58% were males, and 39% had multiple traumas. The frequency of minimal/mild TBI (complicated mild TBI) was 404/830 (49%), moderate TBI (247/830) (30%) and severe TBI 178/830 (21%). Thirty percent of the patients were transported directly from scene of accident to OUH, 39% had primary assessment at local hospital, and 31% had primary assessment at the downtown Oslo Emergency Department. Further patient characteristics are given in Table 2.

**Table 2:** Baseline characteristics of the study population.

			**Antiplatelet (mono or dual)**		**Anticoagulation**		**Antiplatelet and anticoagulation**	
**Characteristic**	**Overall, *N* = 830**	**Antithrombotic naive, *N* = 360^a^**	***N* = 267^a^**	***p*-value^b^^,^^c^**	***N* = 172^a^**	***p*-value^b^^,^^c^**	***N* = 31^a^**	***p*-value^b^^,^^c^**
**Age at injury (years)**	76 (71, 83)	73 (69, 79)	77 (71, 85)	<0.001	81 (74, 86)	<0.001	75 (72, 81)	0.10
**Sex**				0.2		0.13		0.008
Female	345 (42%)	165 (46%)	108 (40%)		66 (38%)		6 (19%)	
Male	485 (58%)	195 (54%)	159 (60%)		106 (62%)		25 (81%)	
**ASA^d^score**				<0.001		<0.001		<0.001
1. Healthy	103 (12%)	100 (28%)	3 (1.1%)		0 (0%)		0 (0%)	
2. Moderate disease	304 (37%)	158 (44%)	95 (36%)		45 (26%)		6 (19%)	
3. Severe disease	398 (48%)	93 (26%)	160 (60%)		122 (71%)		23 (74%)	
4. Life-threatening disease	23 (2.8%)	7 (2.0%)	9 (3.4%)		5 (2.9%)		2 (6.5%)	
**Living**				<0.001		0.002		0.070
Home – independent	622 (76%)	293 (83%)	187 (70%)		120 (71%)		22 (71%)	
Home - with assistance	147 (18%)	44 (12%)	51 (19%)		43 (25%)		9 (29%)	
Institutionalized	51 (6.2%)	16 (4.5%)	28 (11%)		7 (4.1%)		0 (0%)	
Other	2 (0.2%)	2 (0.6%)	0 (0%)		0 (0%)		0 (0%)	
**High energy trauma**				0.029		0.003		0.039
No	653 (80%)	261 (75%)	217 (83%)		147 (86%)		28 (93%)	
Yes	159 (20%)	88 (25%)	46 (17%)		23 (14%)		2 (6.7%)	
**Rotterdam CT score**				0.12		<0.001		0.11
$\frac{1}{2}$	226 (27%)	107 (30%)	71 (27%)		40 (23%)		8 (26%)	
3	431 (52%)	195 (54%)	149 (56%)		72 (42%)		15 (48%)	
4	89 (11%)	23 (6.4%)	29 (11%)		31 (18%)		6 (19%)	
5/6	84 (10%)	35 (9.7%)	18 (6.7%)		29 (17%)		2 (6.5%)	
**HISS^e^**				0.091		0.10		0.3
Minimal/mild	404 (49%)	173 (48%)	143 (54%)		74 (43%)		14 (45%)	
Moderate	247 (30%)	106 (30%)	83 (31%)		45 (26%)		13 (42%)	
Severe	178 (21%)	80 (22%)	41 (15%)		53 (31%)		4 (13%)	
**Multitrauma**				0.047		0.077		0.011
No	504 (61%)	199 (55%)	170 (64%)		110 (64%)		25 (81%)	
Yes	325 (39%)	160 (45%)	97 (36%)		62 (36%)		6 (19%)	

*Two patients had incomplete information on antithrombotics, thus the table gives information on 830 patients*.

a *Statistics presented: median (IQR); n (%)*.

b *Statistical tests performed: Wilcoxon rank-sum test; chi-square test of independence; Fisher's exact test*.

c *P-value calculated against Antithrombotic naive group*.

d *ASA score: The American Society of Anaestesiologists score*.

e *HISS, head injury severity scale*.

### Preinjury Antithrombotic Therapy

Preinjury use of antithrombotic medication was registered in 471/832 (55.6%) patients. Antiplatelet therapy alone (including dual antiplatelet therapy, *n* = 26) was used by 268/832 (31.2%) patients, anticoagulation alone by 172/832 (20.7%), and combined antiplatelet therapy and anticoagulation by 31/832 (3.7%). Acetylsalicylic acid was the most frequently used antiplatelet. Anticoagulation was used in 203/832 (24.4%) patients; VKA in 83, DOAC in 102, and LMWH in 18. Patients using antithrombotic therapy were more likely to be older, have more comorbidity, and have suffered low energy trauma than antithrombotic naive patients (Table 2). The most common indications for antiplatelet therapy and anticoagulation were CVD prevention (primary or secondary) and AF, respectively. Further information regarding types and indications for antithrombotic therapy are given in Tables 3, 4.

**Table 3:** Indications for the use of platelet inhibitors.

**Antiplatelet indication**	**Overall** ***N* = 298**	**Acetylsalicylic acid (ASA)** ***N* = 246^a^**	**Clopidogrel** ***N* = 19^a^**	**Dipyridamol** ***N* = 7^a^**	**Dual antiplatelet therapy^b^** ***N* = 26^a^**
Secondary prophylaxis (coronary disease/PVD^c^)	129 (43%)	106 (43%)	5 (26%)	1 (14%)	17 (65%)
Secondary prophylaxis (Stroke/TIA^d^)	81 (27%)	54 (22%)	13 (68%)	6 (86%)	8 (31%)
Primary prophylaxis	47 (16%)	47 (19%)	0 (0%)	0 (0%)	0 (0%)
Other	14 (4.7%)	12 (4.9%)	1 (5.3%)	0 (0%)	1 (3.8%)
(Missing)	27 (9.1%)	27 (11%)	0 (0%)	0 (0%)	0 (0%)

a *Statistics presented: n (%)*.

b *ASA + clopidogrel, dipyridamol, tikagrelor, or prasugrel*.

c *PVD, peripheral vascular disease*.

d *TIA, transient ischemic attack*.

**Table 4:** Indications for the use of anticoagulation.

**Anticoagulant indication**	**Overall** ***N* = 203**	**Warfarin** ***N* = 83^a^**	**DOAC^c^** ***N* = 102^a^**	**LMWH^d^** ***N* = 18^a^**
Atrial fibrillation (AF)	154 (76%)	60 (72%)	90 (88%)	4 (22%)
Pulmonary embolism (PE)/VTE^*b*^	25 (12%)	9 (11%)	9 (8.8%)	7 (39%)
Mechanical heart valve	8 (3.9%)	8 (9.6%)	0 (0%)	0 (0%)
Thrombosis prophylaxis	8 (3.9%)	3 (3.6%)	0 (0%)	5 (28%)
Other	3 (1.5%)	2 (2.4%)	1 (1.0%)	0 (0%)
Acute coronary syndrome	2 (1.0%)	0 (0%)	0 (0%)	2 (11%)
(Missing)	3 (1.5%)	1 (1.2%)	2 (2.0%)	0 (0%)

a *Statistics presented: n (%)*.

b *VTE – venous thromboembolism*.

c *Direct oral anticoagulants*.

d *Low molecular weight heparin*.

### Antithrombotic Therapy in Study Patients Compared to the General Norwegian Population

**Figure 1:**
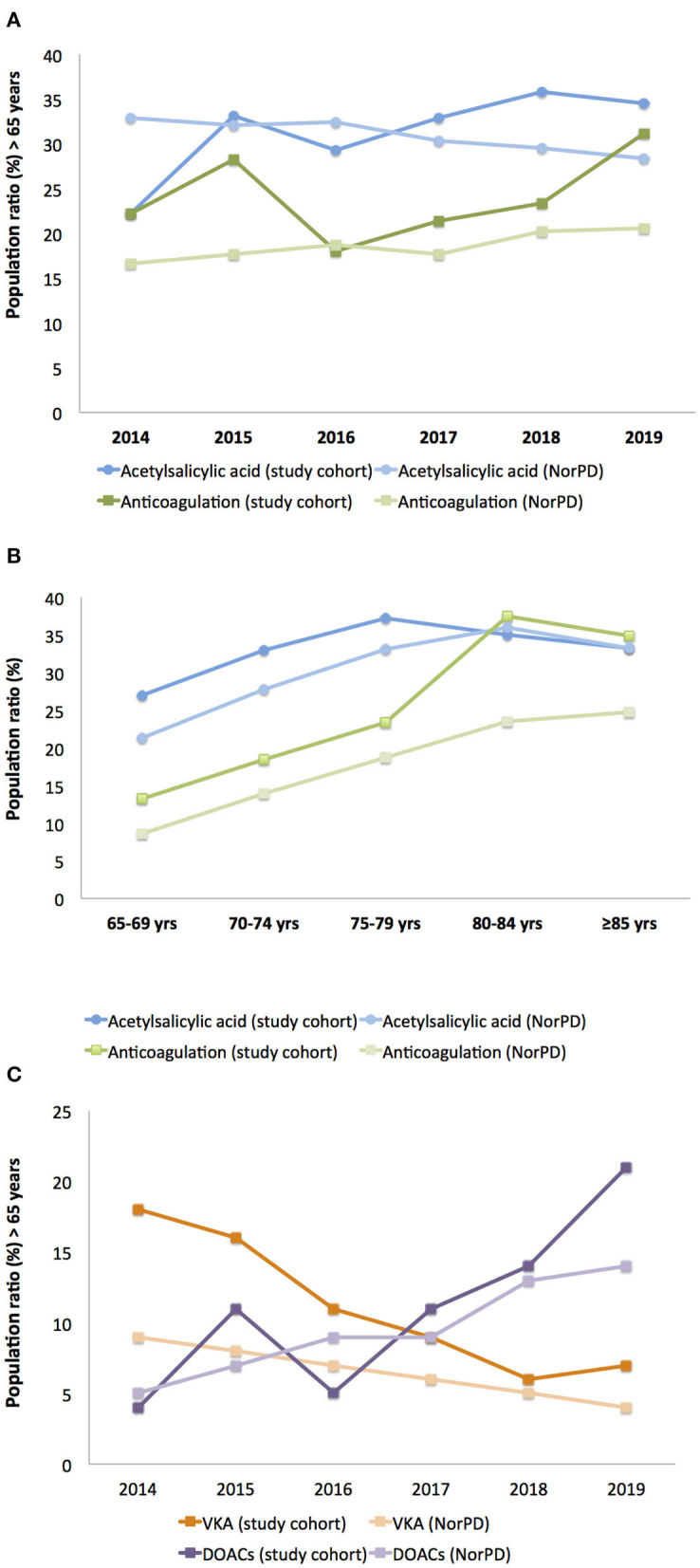
Antithrombotic therapy in study patients compared to the general Norwegian population. **(A)** Use of acetylsalicylic acid and anticoagulation, **(B)** use of acetylsalicylic acid and anticoagulation stratified by age groups, **(C)** use of VKA and DOACs. VKA: Vitamin K antagonists. NorPD: The Norwegian Prescription Database. DOACs: Direct Oral Anticoagulants.

Acetylsalicylic acid was the most used antiplatelet therapy in the study cohort. The frequency of acetylsalicylic acid use did not differ between the study cohort and the general Norwegian population ≥65 years (31 vs. 31%, *p* = 0.87) (Figure 1A). The study cohort used anticoagulation therapy more commonly than the general Norwegian population (24 vs. 19%, *p* = 0.04) (Figure 1A). As outlined in Figure 1B, the use of antiplatelet and anticoagulant therapy both increased with increasing age in the study cohort and the general Norwegian population. However, compared with the general Norwegian population, a higher frequency of patients ≥80 years used anticoagulation in the study cohort (36 vs. 24%, *p* = 0.04). During the study period, the use of VKA declined in parallel with the increasing use of DOACs, both in the study cohort and in the general population (Figure 1C).

### 30-Day Mortality

Overall 30-day mortality was 151/832 (19%). Univariate logistic regression found increasing age, preinjury ASA score ≥3, dependent living, Rotterdam CT-score ≥4, moderate/severe head injury, anticoagulant therapy, and combined antiplatelet and anticoagulant treatment significantly associated with increased 30-day mortality (Table 5). In multivariate logistic regression, increasing age, male sex, dependent living, Rotterdam CT-score ≥4, moderate/severe head injury, and combined use of antiplatelet and anticoagulant therapy remained significantly associated with increased 30-day mortality (Table 5). We obtained identical results for 60-, 90-, and 180-day mortality (data not shown). A separate logistic analysis investigating the association between the different anticoagulants and 30-day survival was also calculated and showed no difference in 30-day mortality between patients using VKA, DOACs, or LMWH [compared to VKA in multivariate analyses: DOACs had OR 0.57 (95% CI 0.22–1.47, *p* = 0.25) and LMWH had OR 1.19 (95% CI 0.25–5.08, *p* = 0.82)].

**Table 5:** Logistic regression for 30-days outcome.

		**Outcome at 30 days**		**Univariate**			**Multivariate**		
**Characteristic**	***N***	**Alive (*N* = 679)^a^**	**Dead (*N* = 151)^a^**	**OR^b^**	**95% CI^b^**	***p*-value**	**OR^b^**	**95% CI^b^**	***p*-value**
**ASA^c^ score**	828								
1. Healthy		92 (14%)	11 (7.3%)	—	—		—	—	
2. Moderate organic disease		260 (38%)	44 (29%)	1.42	0.72, 2.99	0.33	1.29	0.48, 3.69	0.62
3. Severe organic disease		308 (45%)	90 (60%)	2.44	1.30, 5.02	0.009	1.71	0.61, 5.17	0.32
4. Life-threatening organic disease		18 (2.7%)	5 (3.3%)	2.32	0.67, 7.26	0.16	0.92	0.16, 4.99	0.93
**Living**	822								
Home—without assistance		529 (78%)	93 (63%)	—	—		—	—	
Home—with assistance		110 (16%)	37 (25%)	1.91	1.23, 2.93	0.003	2.93	1.52, 5.71	0.001
Institutionalized		35 (5.2%)	16 (11%)	2.60	1.35, 4.82	0.003	2.70	1.09, 6.53	0.029
Other		1 (0.1%)	1 (0.7%)	5.69	0.22, 145	0.22	7.12	0.27, 192	0.18
**High energy trauma**	812								
No		531 (79%)	122 (85%)	—	—		—	—	
Yes		137 (21%)	22 (15%)	0.70	0.42, 1.12	0.15	0.91	0.42, 1.91	0.81
**Rotterdam CT score**	830								
$\frac{1}{2}$		209 (31%)	17 (11%)	—	—		—	—	
3		388 (57%)	43 (28%)	1.36	0.77, 2.51	0.30	1.04	0.54, 2.05	0.92
4		61 (9.0%)	28 (19%)	5.64	2.93, 11.2	<0.001	1.81	0.77, 4.34	0.18
5/6		21 (3.1%)	63 (42%)	36.9	18.8, 76.4	<0.001	11.3	4.70, 28.3	<0.001
**HISS^d^**	829								
Minimal/mild		389 (57%)	15 (9.9%)	—	—		—	—	
Moderate		209 (31%)	38 (25%)	4.72	2.59, 9.03	<0.001	5.44	2.73, 11.4	<0.001
Severe		80 (12%)	98 (65%)	31.8	18.0, 59.6	<0.001	25.7	12.0, 58.5	<0.001
**Multitrauma**	829								
No		417 (62%)	87 (58%)	—	—		—	—	
Yes		261 (38%)	64 (42%)	1.18	0.82, 1.68	0.38	1.65	0.95, 2.86	0.073
**Sex**	830								
Female		289 (43%)	56 (37%)	—	—		—	—	
Male		390 (57%)	95 (63%)	1.26	0.88, 1.82	0.22	1.70	1.02, 2.87	0.043
**Antithrombotic therapy**	830								
Antithrombotic naive		309 (46%)	51 (34%)	—	—		—	—	
Antiplatelet therapy (mono or dual)		228 (34%)	39 (26%)	1.04	0.66, 1.62	0.88	1.10	0.57, 2.12	0.78
Anticoagulant therapy		120 (18%)	52 (34%)	2.63	1.69, 4.08	<0.001	1.60	0.80, 3.19	0.18
Antiplatelet + anticoagulant therapy		22 (3.2%)	9 (6.0%)	2.48	1.03, 5.53	0.032	3.39	1.00, 10.7	0.041
**Age at injury (years)**	830	75 (70, 83)	79 (73, 86)	1.05	1.02, 1.07	<0.001	1.07	1.04, 1.11	<0.001

a *Statistics presented: n (%); median (IQR)*.

b *OR, odds ratio, CI, confidence interval*.

c *ASA score, The American Society of Anaestesiologists score*.

d *HISS, head injury severity scale*.

## Discussion

In this cohort of patients ≥65 years with TBI, 55.6% used antithrombotic medication before the injury. Preinjury antiplatelet therapy was used by 31%, preinjury anticoagulant therapy by 21%, and combined preinjury antiplatelet and anticoagulant therapy by 4%. Preinjury use of antiplatelet therapy was equivalent to the general population's use, while preinjury use of anticoagulant therapy was higher. Multivariate logistic regression analysis did not show any association between preinjury antiplatelet or anticoagulant therapy alone and 30-day mortality. This study did not detect a difference in 30-day mortality between patients using VKA, DOACs, or LMWH.

### Antithrombotic Use in the Study Population and General Population

Acetylsalicylic acid was by far the most used antiplatelet therapy in the study cohort. The frequency of acetylsalicylic acid use did not differ between the study cohort and the general Norwegian population ≥65 years (31% in both), indicating that acetylsalicylic acid alone is not a major factor contributing to the initiation of intracranial bleeding after blunt head trauma. Some previous studies have reported an increased risk of intracranial bleeding for individuals on antiplatelet therapy after blunt head trauma, while most recent studies are in line with our observation (8, 11, 12, 14, 20, 27). Anticoagulation therapy was more frequently used in the study cohort than in the general Norwegian population (24 vs. 19%), particularly in patients ≥80 years, indicating that anticoagulants may contribute to the initiation of intracranial bleeding after blunt head trauma. This observation is in line with several previous studies (8, 11, 12, 17, 20, 27, 28). During the study period, the use of VKA declined in parallel with the increasing use of DOACs, and this is in line with rates reported in the general population.

### 30-Day Mortality

The present study included only hospital-admitted patients ≥65 years with acute TBI identified on neuroimaging. According to HISS, 49% had minimal/mild TBI, 30% had moderate TBI and 21% had severe TBI 178/830. Minimal/ mild TBI with traumatic findings on CT is by some authors referred to as complicated mild TBI (23, 24). The head injury severity and age must be taken into account when comparing our overall 30-day mortality of 19% to other studies. Age and injury severity are closely associated with mortality, and mortality after severe TBI in patients ≥65 years is reported as high as 67% (29). The European CENTER-TBI study recently reported an overall mortality of <10% (30). In the CENTER-TBI study, however, all ages were included. The proportions of moderate and severe TBIs were smaller, and only ~60% had any intracranial abnormality, thus reflecting the general TBI population. In our study of patients ≥65 years, the following factors were associated with increased 30-day mortality; increasing age, male sex, dependent living, Rotterdam CT-score ≥4, moderate/severe head injury, and combined use of antiplatelet and anticoagulant therapy. Except for antithrombotic therapy data, this is in line with most previous reports (18, 29, 30).

### Antiplatelet Therapy and 30-Day Mortality

The benefit of antiplatelet therapy for secondary prevention of myocardial infarction, stroke, and death in patients with established CVD is thoroughly documented with effect sizes of about one third for the reduction in non-fatal myocardial infarction, one quarter for the decline in non-fatal stroke, and one-sixth for the decrease in vascular mortality (2). The net benefit on cardiovascular outcomes is demonstrated to outweigh the countering enhanced risk of major bleeding. However, after blunt head trauma, the impact of antiplatelet therapy has not been investigated directly in these studies. On the other hand, although frequently used in the general population, antiplatelet therapy for primary prevention of CVD is currently not recommended on a general basis due to an uncertain net effect on ischemic vs. bleeding risk (31).

In uni- and multivariate logistic regression analyses, single antiplatelet therapy was not associated with increased 30-day mortality. The number of individuals on double antiplatelet medication was too low for reliable statistical analysis. Several studies, but not all, indicate that single antiplatelet therapy poses a small, but significant risk for intracranial bleeding after blunt head trauma (8, 11, 12, 14, 27). The association reported between single antiplatelet therapy and TBI mortality is divergent; some report a slightly increased risk while others report no increased risk (12, 14–16). The lack of association between antiplatelet therapy and TBI mortality in our study could be a consequence of our protocol for acute reversal of antithrombotic medication in the emergency room, although the PATCH trial actually reported that platelet transfusion *increased* the risk of death or dependence in patients with spontaneous intracranial hemorrhage using antiplatelet drugs (32). It could also be that single antiplatelet therapy itself is not a risk factor for TBI mortality. The potential benefit of antiplatelet reversal must be weighed against any potential negative effect on an individual basis. Recent stent implantation in major coronary vessels (left main stem, left anterior descending artery (LAD), bifurcation stenting) in particular merit careful consideration as thrombotic occlusion of proximal coronary vessels have high mortality rates.

The potential minor risk of bleeding related to single antiplatelet drug use following TBI has significant consequences for patients' triage after blunt head trauma. Triage guidelines/recommendations currently incorporate this minor risk, which results in the frequent use of cerebral-CT and hospital observation in patients ≥65 years with minimal blunt head trauma (GCS 15, no loss of consciousness or amnesia, and no focal neurological signs) (33). However, dual antiplatelet therapy increases the risk of spontaneous and traumatic intracranial bleeding and mortality in TBI patients (8, 12, 27, 34, 35).

### Anticoagulant Therapy and 30-Day Mortality

The impact of anticoagulant therapy on reducing the thrombotic risk in patients with AF, VTE, and prosthetic valve thrombosis in patients with mechanical heart valves—the most frequent indications for anticoagulation in this cohort—is well-documented, and the antithrombotic effect is assumed to surpass the accompanying increased bleeding risk (4, 7, 36). Combined treatment with anticoagulant and antiplatelet therapy in patients with an indication for anticoagulation and acute coronary syndrome is associated with increased bleeding risk. Still, international guidelines recommend this combination (3).

In univariate logistic regression analyses, the use of anticoagulant therapy alone or in combination with antiplatelet therapy was significantly associated with increased 30-day mortality. In multivariate logistic regression analyses, the combined use of antiplatelet and anticoagulant therapy remained significantly associated with increased 30-day mortality, while anticoagulant therapy alone did not. Several previous reports have indicated a significant association between anticoagulant therapy and increased mortality in TBI patients, while some recent publications have questioned this association (12, 15–17, 19, 37). This potential inefficaciousness of anticoagulant therapy on TBI mortality is intriguing and calls for possible explanations. There has been a significant shift in anticoagulant therapy from VKA to DOAC during the previous decade. The risk of major bleeding complications, spontaneous and traumatic, for VKA is well-known. So far, DOACs seem to have reduced risk of major spontaneous bleeding complications compared to VKAs, and some studies report reduced bleeding progression and mortality in TBI patients (12, 28, 37–40). However, a recent report indicated a worse outcome with DOAC compared with VKA in patients with intracranial hemorrhage after TBI (18). In our study, there was no difference in 30-day mortality between VKA and DOAC users. Thus, the low impact of anticoagulation on 30-day mortality appears not to be caused by the transition alone from VKA to DOAC use. Another and possibly more plausible explanation for both the low impact of anticoagulant therapy on TBI mortality and no difference between VKA and DOAC, is our protocol for acute reversal of antithrombotic medication in the emergency room^1^. The beneficial effect of anticoagulation reversal treatment on mortality in patients with intracranial hemorrhage using anticoagulation has recently been demonstrated (41), emphasizing the importance of implementing and comply with such protocols. With the available reversal agents throughout the study period, however, one must assume that the reversal of VKA has been more effective than the reversal of the DOAC effect, especially for direct-acting Xa-inhibitors (42–46). The potential benefit of anticoagulation reversal must be weighed against any potential negative effect on an individual basis. The presence of mechanical heart valves (particularly in the mitral position) merit careful consideration as acute heart valve thrombosis has a high mortality rate.

The combined use of antiplatelet and anticoagulant therapy was the only antithrombotic regime that was significantly associated with increased 30-day mortality in our study. This is in line with previous reports (8, 12, 27, 47). Based on the high frequency of elderly anticoagulant therapy users in this cohort and from the results of the multivariate analyses, anticoagulation alone *and* in combination with antiplatelet therapy must still be regarded as a significant risk factor for intracranial hemorrhage after blunt head trauma and for TBI-related mortality. The inherent risk of anticoagulant therapy for the elderly suffering a blunt head trauma is incorporated into triage guidelines/recommendations (33). This is of particular importance in the future as the elderly population is growing. Appropriate measures and management of older patients with TBI before severe bleeding progression may provide better outcomes and less costly treatments.

### Strengths and Limitations

As the present study reports data from a large Level 1 trauma center in South-Eastern Norway, the included patients will most likely represent the majority of Level 1 trauma hospital-admitted older patients with TBI in need of neurosurgical care. The study concerns a contemporary TBI cohort with protocols for immediate reversal of antithrombotic drugs available and with DOACs as the dominating anticoagulant. As a substantial number of trauma patients initially are triaged at local hospitals in our health region, we have reason to believe that some older adults with comorbidities and severe injuries are omitted from a referral. Moreover, the concordance of medical records with the patients' home medication regimes was unknown. Due to small groups, the comparisons between the different anticoagulant regimes have to be evaluated bearing in mind the risk of type II error. Nevertheless, a differential impact on outcome might be relevant as a large recent publication demonstrated a lower risk of intracranial bleeding for apixaban and dabigatran compared to rivaroxaban (48). The potential difference in outcome based on the availability of complete vs. partial reversal (complete reversal available for VKA and one DOAC, partial reversal available for the other DOACs) was also not assessed. Importantly, we have a standard protocol for reversal of antithrombotics in the emergency room. In this study, we have not examined the compliance to this protocol on an individual level, nor have we studied laboratory values for antithrombotic monitoring or potential complications as thromboembolisms secondary to antithrombotic reversal. New studies on antithrombotics and TBI have been started, which include information on antithrombotic reversal at an individual level, laboratory monitoring of coagulation and platelet function, and possible side effects of antithrombotic reversal. Last, we accessed the Norwegian prescription Registry through a public link. It was impossible to sort out the number of persons on dual antiplatelet therapy or combined antiplatelet/anticoagulant treatment through this access. Thus, comparisons of these combinations of antithrombotic therapy between the study group and the general population were not performed, and we limited the comparisons to “use of antiplatelet drugs” and “use of anticoagulants.”

### Conclusions

In this contemporary cohort of TBI patients ≥65 years, neither antiplatelet therapy nor anticoagulant therapy alone was associated with increased 30-day mortality. A higher frequency of patients using anticoagulant therapy than the general Norwegian population may indicate that anticoagulation might contribute to the initiation of intracranial bleeding after blunt head trauma—although potential confounding factors such as comorbidity were not taken into account. No difference between VKA and DOACs was encountered, while combined antiplatelet and anticoagulant therapy posed an increased risk of 30-day mortality.

## Data Availability Statement

The raw data supporting the conclusions of this article will be made available by the authors, without undue reservation.

## Ethics Statement

The studies involving human participants were reviewed and approved by The Data Protection Officer (DPO) at OUH (approval number 2017/3904). Written informed consent for participation was not required for this study in accordance with the national legislation and the institutional requirements.

## Author Contributions

PR did the statistical analyses, while RB and MM retrieved data on antithrombotic use in the study participants. All authors participated substantially in literature search, study design, data interpretation, writing, and critical revision of the manuscript.

## Conflict of Interest

The authors declare that the research was conducted in the absence of any commercial or financial relationships that could be construed as a potential conflict of interest.

## Footnotes

^1^Oslo University Hospital Trauma Manual. www.traumemanualen.no. Accessed June 23, 2020.

^2^A language and environment for statistical computing. R Foundation for Statistical Computing V, Austria. https://www.R-project.org/. Accessed September 30 2020.
